# Worker Perspectives on COVID-19 Risks: A Qualitative Study of Latino Construction Workers in Oakland, California

**DOI:** 10.3390/ijerph19169822

**Published:** 2022-08-09

**Authors:** Erika Meza, Leslie Giglio, Ana O. Franco, Elizabeth Rodriguez, Laura Stock, John Balmes, Jacqueline M. Torres, Alicia Fernandez

**Affiliations:** 1 Department of Epidemiology and Biostatistics, University of California San Francisco, San Francisco, CA 94158, USA; 2 Department of Medicine, Division of General Internal Medicine, Zuckerberg San Francisco General Hospital, University of California, San Francisco, CA 94110, USA; 3 Department of Biology, San Francisco State University, San Francisco, CA 94132, USA; 4 Labor Occupational Health Program, School of Public Health, University of California, Berkeley, CA 94720, USA; 5 Division of Occupational and Environmental Medicine, School of Medicine, University of California, San Francisco, CA 94143, USA; 6 Division of Environmental Health Sciences, School of Public Health, University of California, Berkeley, CA 94704, USA

**Keywords:** COVID-19, occupational health, essential workers, construction workers, Latino health, immigrant health

## Abstract

Latino construction workers in the U.S. have faced a disproportionate risk for COVID-19 infection in the workplace. Prior studies have focused on quantifying workplace risk for COVID-19 infection; few have captured workers’ experiences and perspectives. This study describes COVID-19-related workplace risks from the perspectives of Latino construction workers. We conducted a qualitative study using semi-structured phone interviews with Latino construction workers from the Fruitvale District of Oakland, California. Twenty individuals were interviewed from December 2020 to March 2021. Nearly all participants (19/20) were Spanish-speaking men; mean age 42.6 years. The majority were low-income and over one-third did not have health insurance. Participants worked in varied construction-related jobs ranging from demolition to office work; additionally, four were day laborers, and three belonged to a labor union. We identified four major themes with public health policy and workplace safety implications: (1) Major concern about the risk of SARS-CoV-2 infection for family health and economic wellbeing; (2) Clarity about mask use and social distancing but not disclosure; (3) Variability in access to additional resources provided by employers; and (4) Uncertainty around structural support for SARS-CoV-2 quarantine/isolation. Our findings provide further evidence from workers’ own perspectives of the major gaps experienced during the pandemic in workplace protections and resources.

## 1. Introduction

The COVID-19 pandemic has disproportionately impacted people in the essential workforce [1,2,3]. Despite state and local regulations intended to protect essential workers, those working outside the home during the pandemic have faced disproportionate risk for SARS-CoV-2 infection and severe COVID-19 illness [4,5]. Much of the existing research on COVID-19 workplace risks has focused on essential workers in the healthcare and food industry and less is known about workers in the construction industry [3,4]. Nevertheless, construction workers had one of the highest COVID-19 prevalence rates in the U.S., and multiple studies suggest that construction workers faced high rates of excess mortality during the first year of the pandemic [6,7,8,9]. Notably, one study based in Texas showed that construction workers were five times more likely to be hospitalized with COVID-19 than workers in all other industries [9].

While Latinos constitute 18% of the US population, they are overrepresented in the construction sector, accounting for 30% of the construction workforce nationally [10]. In California, Latinos constitute 39% of the population and 60% of the construction workforce. Latinos made up 56% of COVID-19 cases and 47% of deaths statewide as of December 2020 [11]. Prior to the COVID-19 pandemic, workplace risks and work-related health conditions within the construction industry had been well documented, particularly among Latinos and day laborers [12,13,14]. Structural racism and anti-immigrant policies contribute to a greater susceptibility to poor workplace health conditions among immigrant and migrant workers, including abuses of worker’s rights such as wage theft and dangerous working conditions [15,16]. Particularly among immigrant workers with limited English proficiency, a lack of information regarding worker safety and limited access to institutional resources (e.g., legal representation) contribute to a heightened vulnerability to exploitation [14,17]. As a subset of the broader construction sector, day laborers are particularly vulnerable to increased workplace risks, as they are often undocumented immigrants and at a greater risk for discrimination, persecution, psychological distress, and overall abuses of worker’s rights [18,19]. Throughout the pandemic, pre-existing workplace risks were exacerbated, as Latino frontline workers were overrepresented in low-wage occupations, such as the construction and food sector, which were less likely to have adequate COVID-19 protections [20]. Despite the clear indications of exacerbated infection, hospitalization, and mortality risk among Latino construction workers, it remains unclear what is driving this heightened risk [8,21,22].

In this study, we address that gap by aiming to understand workplace risks of COVID-19 from the perspectives of Latino construction workers. We focused specifically on construction workers living or residing in the Fruitvale community of Oakland, California, a community with one of the highest COVID-19 infection rates in the San Francisco Bay Area. In October 2020, the Fruitvale community had a case rate twice that of the city of Oakland overall, and over three times higher than the rest of Alameda County (4222 vs. 1357 case per 100,000 people) [23]. At a community testing event in the Fruitvale community in September 2020, Latinos in Fruitvale were found to have a COVID-19 positivity rate nine times higher than non-Latinos (4.5% vs. 0.5%) [24]. By characterizing construction worker’s attitudes, behaviors, and experiences regarding COVID-19 at work, we aimed to identify factors that may exacerbate or mitigate risk and inform future workplace policy.

## 2. Materials and Methods

### 2.1. Study Participants

A convenience sample of Latino construction workers was selected from adults who participated in a free, community-based SARS-CoV-2 testing event in the Fruitvale community of Oakland, California, in September 2020. The two-day testing event was implemented through a coalition of community-based organizations (CBOs), the University of California, San Francisco (UCSF), and local public health authorities. Data on SARS-CoV-2 infection among participants at this testing event have been reported elsewhere (15).

All adults tested at the event were asked to complete a survey with sociodemographic and occupational data and asked if they wished to be contacted for future research purposes. Following the event, those who agreed to be contacted for future research and met the following criteria were contacted by phone and invited to participate: (1) 18 years of age or older; (2) identified as Latino/a; (3) English or Spanish speaker; and (4) identified as a construction worker or day laborer working in construction. Based on these criteria, we reached out to 31 potential participants after the testing event. Of these, 5 individuals were deemed ineligible due to changes in occupation or were Mayan Mam speakers and unable to complete an interview in English or Spanish. Additionally, 3 individuals declined to participate and 3 could not be reached. Twenty Latino construction workers agreed to participate. Verbal informed consent was obtained from all participants. Study protocols were approved by the Institutional Review Board at UCSF.

### 2.2. Data Collection

Data collection instruments included a semi-structured interview guide with open-ended questions focused on participants’ workplace-related worries about perceived risk for COVID-19, behavioral changes at job sites since the pandemic onset, and personal experience with COVID-19. The interview guide was developed by COVID-19 researchers with additional input from occupational and environmental health experts (see Supplementary Materials, Supplemental Text for interview guide).

Recruitment and interviews were conducted via phone in English or Spanish between December 2020 and March 2021. Interviews were conducted by bilingual interviewers and lasted 20–30 min. Interviews were audio recorded and transcribed *verbatim*. Following each interview, data collectors wrote summary memos reflecting on what had been learned from each interview. These memos contained the interviewers’ impressions about participant experiences and were used to capture initial thoughts and possible themes.

### 2.3. Data Analysis

Interview transcripts were anonymized and imported into Dedoose Version 8.0.35 (SocioCultural Consultants LLC., Los Angeles, CA, USA), a qualitative data analysis application. Codes and themes were identified by two investigators (EM and LG) in accordance with the principles of grounded theory and a thematic analysis approach [25]. This process involved identifying initial concepts inductively from summary memos, audio recordings, and transcripts. Next, the team grouped similar concepts into themes and subthemes to develop a coding scheme guided by the literature and interview guide. Each transcript was then independently coded by two authors (EM, LG, and ER) using Dedoose to code quotations within transcripts, indicate patterns that emerged from the interviews, and refine the coding scheme. After independently coding transcripts, EM, LG, and ER reviewed all coding for discrepancies and worked together to reach consensus on final coding. Investigator triangulation from multiple coders increased the analytic reliability (interrater kappa coefficient: 0.83, indicating high agreement) and ensured that themes were representative of all conducted interviews.

## 3. Results

We conducted 20 phone interviews with Latino construction workers, 19 were conducted in Spanish. The sample characteristics of all participants are available in Table 1. The mean age was 42.6 ± 9.2 years with 95% of the participants being male. The mean household size was 4.1 ± 1.3; all participants reported speaking Spanish at home and some also reported speaking English (20%) or Mayan Mam (15%). The majority of participants (80%) reported an annual household income <USD 50,000 and almost half reported no health insurance. Participants worked in various construction-related jobs, ranging from general construction to demolition to office work. One-fifth of participants identified as day laborers working informally in general construction and 20% of all participants were members of a labor union.

Our analysis identified four themes describing COVID-19-related perspectives and experiences of Latino construction workers in the workplace: (1) Major concern about the risk of SARS-CoV-2 infection for family health and economic wellbeing; (2) Clarity about mask use and social distancing but not disclosure; (3) Variability in access to additional resources provided by employers; and (4) Perceptions related to structural support for SARS-CoV-2 quarantine and isolation.

### 3.1. Major Concern about the risk of SARS-CoV-2 Infection for Family Health and Economic Wellbeing

Nearly all participants reported concerns regarding the risk for SARS-CoV-2 infection in the workplace, “*Us older adults worry more. We take seriously what is happening right now, especially parents who have children*”. Participants primarily expressed their worry in terms of potential health impacts on their family, “*I have my family, and I wouldn’t like anything like [COVID-19] to happen to them*”, as well as the potential consequences of infection for economic stability “*as bills are always piling up, one worries for oneself and for one’s family*”. One participant who identified as a day laborer clearly stated his concern about the potential consequences of having to take time off from work, “*What worries me, hopefully I don’t catch [COVID-19] because we need a lot… we have to work every day to bring food to the children, all of that. That’s one worries about*”. Participants also mentioned their families as the primary motivating factor around taking precautions at work, as one general construction worker shared, “*I wear my mask and I have disinfectant and all that…nobody is around me, but I do that, I have to do it for my children*”. Only one participant—the sole individual working in an office setting—explicitly expressed no concerns about the risk of SARS-CoV-2 infection at work, “*I feel pretty safe where I’m at right now…they have put in the safety precautions that not all the companies are doing*”.

### 3.2. Clarity about Mask Use and Social Distancing but not Disclosure

#### 3.2.1. Mask Use and Social Distancing

All participants reported their awareness of and compliance with mask use and social distancing as COVID-19 preventive measures in the workplace. For most participants, these measures had become mandatory at their workplace and reinforced through signage, employee supervision, and coworker role-modeling. As an iron worker explained, “*it is mandatory to wear a mask, if not, they call you out or take you out of work*”. A few participants described wearing masks all day as something they were already used to, for example, one demolition worker stated, “*It hasn’t been difficult for us because the eight hours that we mostly work we always have full face [masks] because that way we don’t have accidents for our eyes or we don’t have accidents for our face, because of the type of work that we have*”.

A few participants noted challenges with mask use, particularly while doing strenuous work, sweating and ‘needing to get air’ but otherwise wearing their mask all day. Social distancing had also become normalized for most participants while working, “*our boss has us avoid each other, they have us working separately in certain areas*”, and during breaks, when the majority reported eating alone in their car. Participants understood that by taking these measures, they were ‘doing their part’ to protect themselves and their coworkers, “*everyone has to set the example, taking care of oneself and others*”.

Many participants also noted that social distancing created a loss of the workplace experience. As one construction worker in demolition stated, “*we are not eating together, we are not talking, we are not in meetings [together] as it used to happen*”. Participants described how mealtimes previously served as spaces for social connection, where coworkers would also share meals. Now, many reported total seclusion, eating alone in their car. One participant reported, “*we can’t all be there gathered during breaks or eating together having lunch with other coworkers, no, each of us needs to be in our own space or corner*”. One participant who identified as a day laborer noted, “*one no longer shares like before*”, expressing a loss of comradery and workplace cohesion*, “coworkers would share tools, sometimes one would carry theirs and share it with others, and also pass time with [during breaks] one would like to share a taco*”.

#### 3.2.2. Disclosing COVID-19 Symptoms and Diagnosis

In contrast to the universal clarity around masking and distancing guidelines, there were varying levels of understanding regarding workplace protocols for disclosing symptoms and COVID-19 diagnosis. Most participants did not have personal experience with COVID-19 infection at their workplace at the point of our interviews but expressed they would disclose to their employers if they had symptoms to avoid infecting others. One participant, working in a larger company, reported how strict their employer was about disclosing symptoms, “*Right now, it’s zero tolerance for anyone who comes to work with COVID and puts others at risk*”. Additionally, two participants explicitly shared that their boss was very supportive and encouraged them to be transparent if they felt sick or tested positive to avoid putting the entire group at risk.

### 3.3. Variability in Access to Additional Resources Provided by Employers

#### 3.3.1. Daily Health Screenings

While nearly all participants described using masks and following social distancing protocols, there were marked differences in the few participants that reported access to masks and additional resources through their employer. For example, workers in larger construction companies (five or more coworkers) were more likely to report personal protective equipment (PPE) such as masks and hand sanitizer or hand washing stations provided by their employer. In comparison, most day laborers and construction workers in smaller, less structured, workplaces (i.e., 10 or fewer employees) expressed that they were responsible for providing their own, “*They give us information, yes, but for masks, everyone has to buy their own*”. One participant who identified as a day laborer shared how his different employers never mentioned anything about COVID-19, “*Never, the subject is not even touched upon*”.

Similarly, only participants who worked with larger companies mentioned temperature checks and daily health screening questionnaires, either through the employer or the job site manager. As one demolition worker explained, “*We have to take our temperature when we come in for work, every day as soon as you get there you sign in…it is implemented for the company, we have to fill out a paper every day that we have not been with another person who has been infected, that we do not have a positive person at home*”.

#### 3.3.2. SARS-CoV-2 Testing

Even fewer participants reported having SARS-CoV-2 testing or information on available testing sites through their employer. Most participants reported that their employers did not have the proper resources for them to be tested regularly, with some noting the burden of finding testing sites on their own. For example, one construction worker without health insurance expressed difficulty navigating the healthcare system, “*It’s been about 22 days since I called [a local clinic], but they didn’t answer… and now I haven’t had another [COVID-19] test*”. Only two participants reported that their employer provided testing. One of these participants, who was the only worker in an indoor office setting for a construction company, shared that his employer would set up appointments for employees to get tested. The other participant, a general construction worker at a school, reported taking weekly tests covered by his employer.

#### 3.3.3. Unions as Uncommon Providers of Prevention Information

The majority of participants (17/20) reported that they did not belong to a union. Namely, no day laborers reported being part of a union. While immigration status was not asked of any of our participants, one day laborer specifically noted his immigration status as a barrier for being in a union. Among the three unionized workers, union support for workplace COVID-19 prevention was mixed. Two of them reported not having received any information regarding COVID-19 nor additional resources from their union, while the third reported receiving only minimal educational support, “*The only thing they have told us is that we need to prevent, that we need to use masks*”.

### 3.4. Uncertainty around Structural Support for SARS-CoV-2 Quarantine and Isolation

#### 3.4.1. Fear around the Consequences of COVID-19 Diagnosis Disclosure

Although all participants expressed that they would disclose a positive COVID-19 diagnosis to their employer, there remained uncertainty and concerns about how this would affect their job, especially among participants in smaller workplaces. Only two participants had experienced a positive diagnosis and three additional participants reported having a coworker who tested positive and had disclosed their diagnosis to their superior. Among the two participants who had experienced SARS-CoV-2 infection, a demolition worker reported intense worry about the consequences of disclosure, “*It was a bit like, ‘what is going to happen to me, will they lay me off, will they scold me, what is going to happen, am I doing good or bad?’*”. This concern was also common among those who had not personally experienced SARS-CoV-2 infection. One participant expressed uncertainty about how their boss would react under the hypothetical scenario of reporting a positive diagnosis, “*I don’t know how he would react. We have had no cases of that. None of the coworkers, no one has experienced that*”.

#### 3.4.2. Taking Days off without Pay

The two participants who had tested positive for SARS-CoV-2 by the time of our interviews—both non-day laborers—noted either a complete lack of, or inadequate, paid sick leave. The first participant, shared that the lack of benefits, including paid sick leave, was “*one of the few big problems*” in his company of three workers. The second participant, a demolition worker who reported working for a larger company and who tested positive, described their need to self-advocate before receiving any paid leave, “*After [being out sick] for two months [without pay], I talked to the safety person, and they paid me one week*”.

Only two other participants, a unionized iron worker and a construction worker at a school, expressed with certainty that their employer provided paid leave for COVID-19 positive cases in their workplace. Other participants were unsure about whether they would receive pay beyond their 3 annual sick days, or any other type of supplemental income if they had to take time off work due to COVID-19. As one participant shared, “*Supposedly the law says yes [we get paid sick leave], but I couldn’t tell you that, it hasn’t happened to me, nor to any of my coworkers*”. Another participant also shared what they had heard from their coworkers and at a local community clinic, “*What I do know, more or less, are those [programs] helping people pay the rent, for example in the city of Oakland*”.

## 4. Discussion

We conducted a qualitative study aimed at understanding the workplace risks of COVID-19 from the perspectives and experiences of Latino construction workers in a highly impacted area of Oakland, California. We found that participants universally described clearly understanding and following public health messaging around mask use and social distancing at work. In marked contrast, participants expressed uncertainty and concern about workplace protocols in the event of a positive COVID-19 diagnosis and often felt poorly supported in obtaining testing or other structural support from their employer. Although participants expressed that they would disclose their symptoms or positive test result to their employers, many expressed hesitance and concern about what the potential consequences of a positive test might be. Most participants were uncertain about financial supports including unemployment, paid leave, or local efforts to support mandatory isolation. Those who had experienced COVID-19 shared multiple challenges they encountered, including losing pay and fearing job loss.

Our results are broadly in line with findings from other studies focused on essential workers. A recent survey of Asian and Latino workers in domestic health care and janitorial/hospitality services in California found that half of respondents were concerned about not being able to provide financially for themselves or their families if they became sick [26]. In addition, over half of the participants either did not receive any information from employers about their right to paid sick leave, or received misleading or incomplete information, and did not know if they could use paid sick leave for COVID-19. Lack of information about work benefits and specifically sick leave has also been noted by other essential workers [27]. Although limited access to worker resources and information on standard safety procedures has been longstanding among low-wage, immigrant workers [28], the COVID-19 pandemic has elevated the need for occupational safety and health agencies to develop the institutional capacity to reach groups of workers that have been historically underserved and at increased risk for adverse work-related health outcomes.

### 4.1. Policy Landscape

Our study has important implications for policy and programs related to workplace protections, including the need for improving resources supplied to workers in low-wage essential industries related to PPE, testing, and sick leave. In December 2020, just as our interviews were beginning, the California Occupational Safety and Health Standards Board approved the COVID-19 Prevention Emergency Temporary Standards (ETS) on infection prevention. The ETS, as passed in 2020, called for employers to provide workers with training on COVID-19 prevention techniques, as well as information on COVID-19-related benefits [29]. In addition, employers became required to provide sick leave to COVID-19 positive workers and to notify and support other workers for testing in the event of workplace outbreaks. Workers who were exposed to SARS-CoV-2 at work were required to quarantine for at least 10 days with full pay and benefits. Since 2020 the California ETS has been modified a number of times with required provisions changing as the COVID-19 landscape has changed. Our results support that clear policies setting out requirements for dealing with COVID-19 in the workplace are essential in ensuring the disclosure of a COVID-19 diagnosis and isolation of an infected worker.

The lack of awareness about work benefits expressed by the participants in our study may have also been driven by the limited resources of small employers to provide sick leave. Federal programs, such as the Coronavirus Aid, Relief, and Economic Security (CARES) Act, provided economic relief and support for American workers and small businesses. However, much of the Fruitvale community, including participants in our study, consists of recent immigrant families with mixed legal immigration status who were excluded from federal assistance and safety net programs. In an effort to help fill gaps in federal relief, local county-level recovery programs provided compensation to essential workers and low-income individuals in high-risk neighborhoods to take time off if they tested positive and did not have COVID-19 employment benefits [30]. However, these funds were only intermittently available as they were dependent on philanthropy. Additionally, while we did not ask directly about the role of worker’s immigration statuses, other studies have noted fear of retaliation due to being undocumented as a significant barrier to testing and applying for government benefits [31].

More recently (10 June 2021), the federal Occupational Safety and Health Administration (OSHA) issued a nation-wide ETS for occupational exposure to COVID-19, which was originally meant to apply to all workers. Yet, this federal ETS only required specific employers in health care sectors to develop and implement strategies for prevention and control of COVID-19 hazards in workplaces, leaving essential workers in other industries, including construction, vulnerable to the ongoing pandemic.

### 4.2. Implications for Practice and Research

As COVID-19 workplace policies evolve and continue to demand increased support from employers, there is a need to ensure these are developed with consideration of small, informal employers in high-risk industries. In addition, such policies should support employers with the resources necessary for policy implementation. This is particularly relevant for Latino immigrant construction workers, since a significant proportion work for small businesses with fewer than 10 employees [28]. Prior to the pandemic, small businesses experienced a disproportionate burden of occupational injuries and deaths, largely related to lack of knowledge and capacity [28]. For example, small businesses are less likely to utilize formal training methods perceived as costly and time-consuming. Therefore, given that Latino immigrant construction workers in small businesses fall into more than one category of increased occupational risk, additional constraints of resources can exacerbate the disproportionate burden placed on this already vulnerable subgroup of essential workers.

Our findings provide further evidence of the need for more worker outreach and education on standardized policies for job protection. Occupational safety and health government agencies should build upon the existing trust and relationships built by community-based organizations and collaborate with them to effectively disseminate information on workers’ rights and the responsibilities of employers with regards to COVID-19 protocols [17]. Our findings also suggest the need for institutional resources to support regular testing and paid sick leave among all essential workers, including those working in the informal sector. To protect the health and safety of workers and encourage compliance among employers, enforcement agencies can also work closely with community organizations to help identify industry-specific, regulatory strategies that are more sustainable [32]. Building relationships with local organizations that workers are familiar with can help overcome language barriers, cultural differences, and mistrust of government institutions that can make it difficult for Latino immigrant workers to access information [33].

### 4.3. Strengths and Limitations

Our study was based on a convenience sample of Latino construction workers based in the city of Oakland, California. The composition of the sample may have been impacted by our sampling strategy. It is possible that the experiences of construction workers in other settings with different public health responses and worker protection environments may have varied substantially. In addition, given the rapid changes of workplace policies and behaviors related to COVID-19, our study is limited to the experience of construction workers in late 2020 and early 2021 and does not reflect the dynamics present at other points throughout the pandemic; for example, perspectives on vaccination. Furthermore, while a few respondents shared their legal status during the interview, information regarding participant immigration status was not asked of participants, as the research team did not wish to promote anxiety within a context of heightened distress during the pandemic. While we ascertained all study participants to be Spanish speakers, who spoke a variation of Spanish, English, and Mayan Mam, we did not collect information regarding participant English proficiency level.

Despite these limitations, the insights from the experiences of construction workers conveyed by our study complement previous quantitative research on the population level risks of COVID-19 and have implications for workplace policies. Our findings support recent workplace policy changes in California and highlight the need for a wider dissemination of information about the current regulations. Our findings also underscore the need for additional resources to be made available to employers and workers to allow more widespread implementation of such policies amongst the most vulnerable workers.

## 5. Conclusions

The COVID-19 pandemic has left construction workers, among other essential workers, disproportionately exposed to SARS-CoV-2 infection and severe COVID-19. Our qualitative study with Latino construction workers provides further evidence from workers’ own perspectives of the major gaps in workplace protections and resources experienced during the pandemic. Our findings support efforts to expand paid sick-leave to all at-risk workers and underscore the need to ensure workers and employers are aware of COVID-19-related mandated workplace policies and resources.

## Figures and Tables

**Table 1:** Sample Characteristics of Latino participants interviewed (N = 20).

	n	%
**Mean Age** (SD)	42.6	(9.2)
**Male**	19	95%
**Mean Household Size** (SD)	4.1	(1.3)
**Language spoken at home**		
Spanish only	13	65%
Spanish and English	4	20%
Spanish and Mam	3	15%
**Annual Household Income**		
<USD 50,000	16	80%
USD 50,000–USD 100,000	1	5%
Prefer not to disclose	3	15%
**Primary Type of Construction Work**		
General ^a^	10	50%
Demolition	3	15%
Painting	3	15%
Ironwork	1	5%
Concrete	1	5%
Roofing	1	5%
Office Worker	1	5%
**Day Laborer** (**in construction**)	4	20%
**Coworkers daily contact**		
None (individual work)	2	10%
2–5 coworkers	14	70%
5 or more coworkers	4	20%
**Belongs to a labor union**	3	15%
**COVID Experience**		
Ever tested positive for COVID-19	2	10%
Ever exposed to COVID-19 case at work	3	15%
**Health Insurance**		
Uninsured	8	40%
Public Insurance	7	35%
Other Private Insurance	4	20%
Prefer not to disclose	1	5%

^a^ Includes more than one type of construction work (excludes day laborers). Demographic and socioeconomic characteristics were collected at the time of the community-based COVID-19 testing event in September 2020.

## Data Availability

Summaries of the data presented in this study are available on request from the corresponding author. The data are not publicly available due to containing potentially identifiable information about the participants.

## Supplementary Materials

The following supporting information can be downloaded at: https://www.mdpi.com/article/10.3390/ijerph19169822/s1, Supplemental Text.
