# Supplementary material for: Worker Perspectives on COVID-19 Risks: A Qualitative Study of Latino Construction Workers in Oakland, California
Source: Int J Environ Res Public Health. 2022 Aug 9;19(16):9822. doi: 10.3390/ijerph19169822 (PMC9408167; doi:10.3390/ijerph19169822)
Supplement: Supplementary file 1 [file ijerph-19-09822-s001.zip › ijerph-1807101-supplementary.pdf]

Supplemental Text. Construction Workers' Experiences in the Workplace Interview Guide

Opening statement:

Thank you for agreeing to participate in a short interview about your experiences during the COVID-19 pandemic. As we discussed, this is part of a scientific study. Everything we discuss in this interview will be kept confidential. Your name will not be used in any of the findings.

Let's start by talking about your job.

1. Could you tell me what kind of work you generally do?
    a. What do you do at work? Is it mainly indoor/outdoor?
    b. Do you work for another person or a company? Or are you self-employed?

2. In general, how many people are at a worksite with you?
    a. How many of these people are from your same crew or company?
    b. In general, how many coworkers do you have close contact with every day at work? What about during breaks?

3. What forms of transportation do you use for getting to/from work? What about for getting to different job sites?
    a. Do you share a ride with the same people or different people every day?
    b. If YES – What do you or others do while you are sharing rides? Are people masked? Do you roll down the windows?

4. At your job, do your coworkers have any worries about their risk for COVID-19? Why or why not?
    a. What do they do or not do that makes you think that?

5. At your job, do you have any worries about your risk for COVID-19? Why or why not?
    a. Do you do anything to lower your risk of contracting COVID-19 at work?

6. Have there been any changes in how you or your colleagues do your work because of COVID-19?
    a. Has your employer done anything to make work safer (e.g., provided trainings, masks)?
    b. How did that change come to be? (Did that come from your employer? From you?)

Now, I want to ask you some more specifics about your workplace since the start of the COVID-19 pandemic.

7. Masking is one way to help protect from COVID-19, but we know it can be hard wearing masks at all times at work. Tell me about mask wearing at your workplace?
    a. How common is it, and what kind of face coverings are used?

    b. Who provides the masks for you and your coworkers?
    c. Are there situations where it is difficult for you or your coworkers to keep your mask on while working?

8. Now let's talk about being physically distant from your coworkers or clients. Are there situations where it is difficult to maintain six feet of distance at work? Why or why not?
    a. What about during breaks?
    b. Has your employer taken steps to make it easier for you to physically distance at work?

Now we want to understand more about the relationship between employees and employers.

9. We know it is not always easy to talk with your employer about personal health. How would you feel about telling your employer if you had been exposed or were experiencing symptoms of COVID-19? Why?
    a. Does your employer do anything to make their workers feel comfortable telling them if they feel sick or if they were exposed?
    b. Does your employer allow their workers to stay home if they have symptoms of COVID-19 or were exposed to COVID-19?

10. What does your employer offer employees that need to take days off due to COVID-19 symptoms or a positive test?
    a. Do they offer paid sick leave or other resources that allow workers to stay home if they were exposed to COVID-19 or have symptoms of COVID-19?
    b. **IF YES:** Could you tell me more? Have you or someone else used these resources?

11. Are you represented by a union?
    a. Have you received any information from the union, or, has the union done anything about COVID-19?
    b. **IF YES:** What exactly have they done related to COVID-19 (resources, activities)?
    c. **IF NO:** Is there another person or resource that you could reach out to if you had questions or concerns about COVID-19?

Now I want to know about your own experience with COVID-19. As before, all questions are confidential and there are no right or wrong answers.

12. Has anyone in your workplace tested positive for COVID-19?
    a. IF YES: Was it somebody at your worksite or apart of your company?

If YES, what happened at your workplace after they tested positive?

    a. How did they find out (i.e., did your employer send them to get tested or did they get tested on their own)?
    b. Were other coworkers notified? Did your employer have others be tested?

c. Were there any changes at your job or disinfecting procedures after they tested positive?
d. Did your employer have specific requirements before you came back to work (e.g., negative test results, 10 days no symptoms, no fever for 24+ hours)?

If NO, do you know what would happen if someone at your job tested positive?

a. Does your employer ask you or your coworkers to be tested for COVID-19?

13. Are there other things you would like your employer to do to protect you at work? (e.g., policies or other resources?)

14. In general, are there activities that you or your coworkers do outside of work that you believe put you at higher risk for COVID-19?

That is all the questions I had for you today. Do you have any questions for me or are there any other important things you'd like to share or add?

Thank you for your time, have a great day!
